# Substrate Materials for Biomolecular Immobilization within Electrochemical Biosensors

**DOI:** 10.3390/bios11070239

**Published:** 2021-07-15

**Authors:** Ian Ivar Suni

**Affiliations:** 1 Materials Technology Center, Southern Illinois University, Carbondale, IL 62901, USA; isuni@siu.edu; Tel.: +1-618-453-7822; 2 School of Chemistry and Biomolecular Sciences, Southern Illinois University, Carbondale, IL 62901, USA; 3 School of Mechanical, Aerospace and Materials Engineering, Southern Illinois University, Carbondale, IL 62901, USA

**Keywords:** biosensor, electrochemistry, immobilization, protein, deoxyribonucleic acid, enzyme, aptamer, antibody, amperometry, electrochemical impedance spectroscopy

## Abstract

Electrochemical biosensors have potential applications for agriculture, food safety, environmental monitoring, sports medicine, biomedicine, and other fields. One of the primary challenges in this field is the immobilization of biomolecular probes atop a solid substrate material with adequate stability, storage lifetime, and reproducibility. This review summarizes the current state of the art for covalent bonding of biomolecules onto solid substrate materials. Early research focused on the use of Au electrodes, with immobilization of biomolecules through ω-functionalized Au-thiol self-assembled monolayers (SAMs), but stability is usually inadequate due to the weak Au–S bond strength. Other noble substrates such as C, Pt, and Si have also been studied. While their nobility has the advantage of ensuring biocompatibility, it also has the disadvantage of making them relatively unreactive towards covalent bond formation. With the exception of Sn-doped In_2_O_3_ (indium tin oxide, ITO), most metal oxides are not electrically conductive enough for use within electrochemical biosensors. Recent research has focused on transition metal dichalcogenides (TMDs) such as MoS_2_ and on electrically conductive polymers such as polyaniline, polypyrrole, and polythiophene. In addition, the deposition of functionalized thin films from aryldiazonium cations has attracted significant attention as a substrate-independent method for biofunctionalization.

## 1. Introduction

According to IUPAC, a biosensor is “a device that uses specific biochemical reactions mediated by isolated enzymes, immunosystems, tissues, organelles or whole cells to detect chemical compounds usually by electrical, thermal or optical signals [1]”. This definition is often assumed to include acoustic signal transduction. Electrochemical signal transduction methods include potentiometry, amperometry, conductometry, electrochemical impedance spectroscopy (EIS), and field effect transistors (FET) [2]. Due to their all-electrical nature, advantages of electrochemical biosensors for signal transduction relative to other methods include simplicity, greater robustness, lower cost, and easier miniaturization and integration into electronic circuits [3,4]. The last advantage is particularly important for real-time integration and communication of biosensors with smartphones [5]. However, to date commercial biosensor technology is limited primarily to glucose detection for diabetes management using both handheld and transdermal devices [6,7], with the worldwide market expected to reach $31 billion in 2022. Lactate biosensors may also be available for applications to food processing, clinical care, and sports medicine [8].

The reasons for the slow introduction of biosensors into the marketplace are complex and depend upon the signal transduction method, biomolecular probes and their immobilization chemistry, complex media (i.e., blood, water, food) that is tested, and the desired detection limit and linear range for a specific application. Practical limitations to the introduction of biosensors include the market need for sensors to detect a specific analyte, advantages of biosensor technology relative to current methods such as enzyme-linked (ELISA) and lateral flow immunoassays, and the cost and ease of manufacturing [9]. Scientific and engineering challenges that have attracted recent interest include non-specific adsorption from complex sample matrices; flexible materials for wearable devices; device miniaturization; incorporation of membrane proteins, nanomaterials and two-dimensional materials; and attaining the exquisite sensitivity needed for detection of DNA [10]. 

One important limiting factor for electrochemical biosensors that bridges practical and scientific challenges is the stability, storage lifetime, reproducibility, and reusability of biomolecules attached to an electrically conductive substrate material, which is the topic of this review. A benchmark for biosensor storage lifetime of at least six months is sometimes quoted [9]. It should be noted that surface immobilization is not always necessary to create an effective biosensor. Glucose oxidase, glucose dehydrogenase and other enzymes employed within glucose biosensors do not usually establish direct electrical contact with the underlying substrate material, but communicate instead through a redox mediator [6]. Nevertheless, direct electrical communication between enzyme and substrate material is still desirable within enzymatic biosensors. For electrochemical biosensors that detect analyte binding, close contact between antibodies, receptor proteins, DNA, or aptamers to the underlying substrate is usually required.

Table 1 summarizes the physical and chemical properties and advantages and disadvantages of the substrate materials considered in this review. These entries can be divided into noble elements such as Au, C, Pt and Si; oxides such as TiO_2_ and Sn-doped indium oxide, otherwise known as indium tin oxide (ITO); sulfides such as MoS_2_ and WS_2_; and electrically conductive polymers. Most metallic elements are active enough to be dissolved or leached out of an electrode and may be biotoxic, so only noble metals have been widely studied for biofunctionalization. Metal oxides may be stable in common sample matrices, but most are not electrically conductive enough for use as sensor electrodes. Although conductive polymers are a significant area of interest, their electrical resistivity is still high, as shown in Table 1. The discussion will start in Section 2 with Au electrodes, which are the historical origin of electrochemical biosensors to which most subsequent efforts refer. 

Many important topics in the field of electrochemical biosensors are outside the scope of this review. For example, recent review articles already discuss the potential applications of biosensors to agriculture [11,12], food safety [11,13,14], environmental monitoring [11,14,15,16], sports medicine [17], chemical and biological warfare prevention [18,19], and healthcare [20,21], including glucose management [6] and early cancer detection [22,23]. In addition, recent review articles have been published on the chemical methods and strategies employed for electrode biofunctionalization [24,25,26,27,28,29,30,31,32,33,34,35,36,37,38]. Variation in sensor performance with biofunctionalization method is often observed [39,40]. 

**Figure 1:**
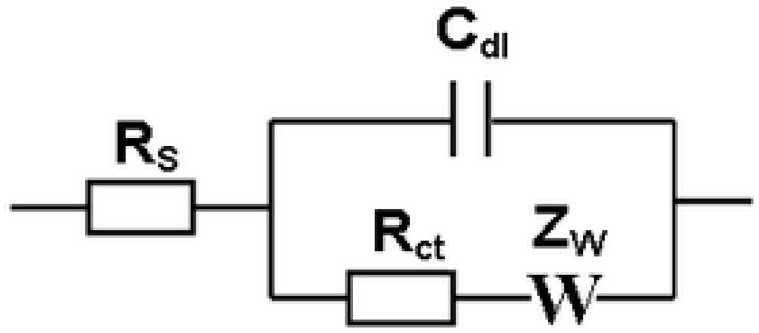
Randles equivalent circuit, where R_S_ is the solution resistance, R_ct_ is the charge-transfer resistance, C_dl_ is the double-layer capacitance, and Z_W_ is the Warburg impedance.

Recent review articles have also been published that describe the electrochemical methods employed for biosensing, including potentiometry [41,42], amperometry [42,43], conductometry [43], electrochemical impedance spectroscopy (EIS) [44,45], and differential pulse voltammetry (DPV) [46]. However, the most widely studied electrochemicalmethods are amperometry, which typically employs oxidoreductase enzymes for biocatalysis, and impedance methods, which may employ antibodies, receptor proteins, deoxyribonucleic acid (DNA), or aptamers for bio-recognition. In the discussion below, sensor stability and storage lifetime for amperometric biosensors are usually monitored by changes in current density. The results from impedance biosensors are usually fit to a Randles equivalent circuit (Figure 1) [47], and the sensor stability and storage lifetime are monitored by changes in the charge transfer resistance (R_ct_), which is discussed further in Section 2.1. R_ct_ typically increases with the biomolecular film thickness and surface coverage, often following a Langmuir adsorption isotherm.

## 2. Au-Thiol Self-Assembled Monolayer (SAM) Formation 

### 2.1. Overview

The “gold standard” for biomolecule immobilization is formation of an Au-thiol self-assembled monolayer (SAM) using a bi-functional linker molecule, with a thiol group at one end and a chemically reactive group at the other end (ω functionalization) for covalent bonding of biomolecular probes [24,48]. Similar results are obtained using a symmetric dithiol compound, where the disulfide bond in the middle is reduced, forming two Au–S surface bonds and exposing two ω-functional groups. Biomolecules can be covalently bound to many reactive groups, including maleimide, amino, alkene, alkyne, azide, and carboxylate groups, as detailed elsewhere [24,25]. The most common ω-functionalization for biosensing atop Au-thiol SAMs are carboxylate groups, which can form an amide bond with amino groups on the surface of a biomolecule using carbodiimide chemistry [49].

Alternatively, thiol groups within biomolecules can be utilized for direct formation of Au-S covalent bonds without an intervening bi-functional reagent or SAM. This has been widely used to immobilize proteins directly onto Au through cysteine residues in the wild-type protein, or through cysteine residues introduced by genetic engineering into otherwise cysteine-free proteins [26,27]. Advantages of this approach include the low prevalence of cysteine residues in natural proteins, which allows control of the immobilization site on the protein surface, as well as the modest impact of cysteine introduction on protein activity. Cysteine residues are often introduced at either the N- or C-terminus, or at positions opposite the active site in antibodies or enzymes [26,27]. Direct binding to glassy carbon through genetically introduced cysteine residues has also been reported [50,51]. However, direct protein attachment through cysteine residues to an Au or C electrode has not been reported to improve sensor stability or storage lifetime.

A variation of this method involves chemical reduction of disulfide bonds in the hinge region of antibodies using reducing agents such as dithiothreitol (DTT) or tris(2-carboxyethyl)phosphine (TCEP), allowing direct formation of Au–S bonds to the resulting antibody fragments [28]. Recently, an increase in sensitivity of ~100× was observed for impedance detection with half-antibodies directly attached to an Au electrode relative to whole antibodies immobilized through amide bond formation to a SAM [52]. This sensitivity improvement was attributed to the increased electron transfer rate without the presence of an SAM, which is electrically insulating. 

DNA probes modified with either six- or 11-carbon thiol linkers can also be directly attached to Au electrodes [29] without an intervening linker chemistry. DNA immobilization is typically followed by addition of a short-chain thiol such as 6-mercapto-1-hexanol (MCH) to form a close-packed film that passivates the Au surface and reduces the background signal [29]. This also reorients the DNA probes somewhat by disrupting non-specific interactions and pushing them away from the surface so that they are tilted at ~30° relative to the surface normal. The use of an 11-carbon thiol linker increases the stability of DNA-based biosensors from about 1 to 7 days [29]. However, the use of 11-carbon chains dramatically slows the electron transfer rate at DNA-coated electrodes relative to six-carbon chains. 

Therefore, a trade-off exists between optimizing sensor stability, which is enhanced by long carbon linker chains as in SAM formation, and optimizing electron transfer rate (and thus sensitivity), which is enhanced by short carbon linker chains. This is illustrated by the variation with film thickness (x) in the charge transfer resistance (R_ct_) through an Au-thiol SAM [53]:

(1) $$R_{\mathrm{ct}}=R_{\mathrm{ct}}^{0}e^{\beta x}$$

where R_ct_^0^ and β are constants, with β typically in the range 0.05–0.11 nm^−1^. Since R_ct_ is reciprocally related to the electron transfer rate constant, Equation (1) predicts an exponential decline in electron transfer rate with increasing film thickness.

**Figure 2:**
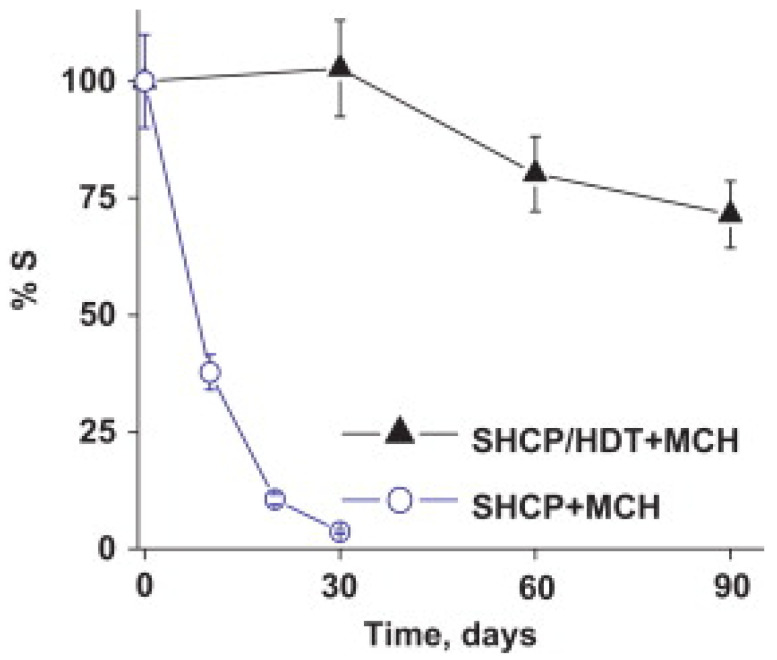
Stability of DNA biointerfaces atop binary and ternary self-assembled monolayers (SAMs), as measured by % retention of the original current response. Reprinted with permission from Ref. [54]. Copyright 2012 Elsevier B.V.

The best results in the literature for stable immobilization of biomolecules through an Au-thiol SAM utilize a ternary SAM that includes thiolated DNA [54,55,56,57,58]. Excellent stability was reported by Wang and co-workers for a ternary SAM formed by first exposing the Au electrode to a binary mixture of a thiolated capture probe and 1,6-hexanedithiol, and then exposing the surface to MCH [54]. Prior to this report, addition of MCH was already widely utilized to disrupt non-specific interactions between DNA and Au, fill the space between DNA molecules, and force thiolated DNA into a more vertical orientation. However, binary SAMs that contain only probe DNA and MCH were found to be unstable, with gradual displacement of probe DNA by MCH. The addition of a third component (dithiol) into the ternary SAM minimizes displacement of probe DNA by MCH [54]. This ternary SAM retained 72% of its sensitivity towards a sandwich-type DNA hybridization assay after 90 days, whereas the sensitivity of a binary SAM without a dithiol declined to 10% after 20 days of storage, as illustrated in Figure 2 [54]. 

**Figure 3:**
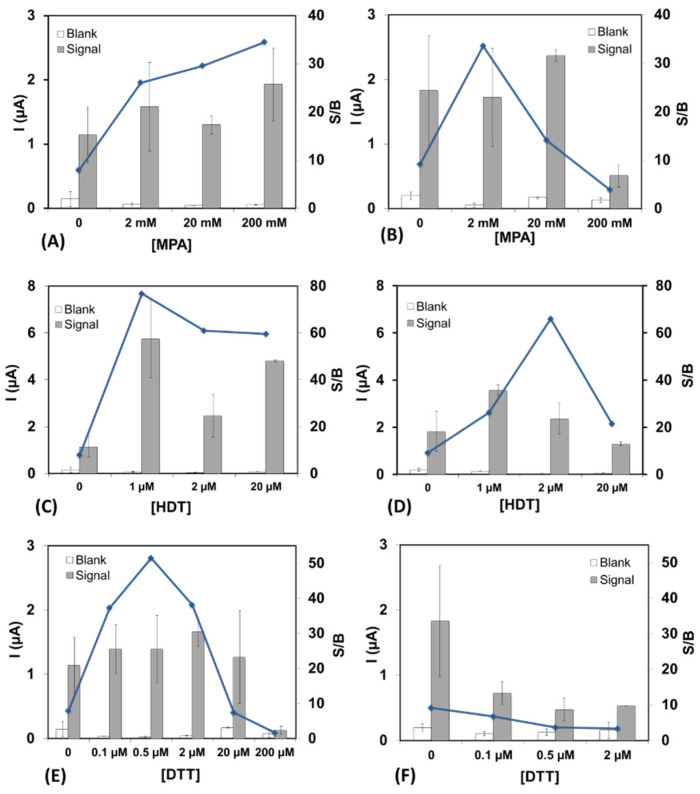
Effect of short-chain diluent concentration on the signal/background (S/B) ratio for gluten (**A**,**C**,**E**) and peanut (**B**,**D**,**F**) genosensors upon addition of increasing concentrations of 3-mercaptopropionic acid (MPA), 1,6-hexanedithiol (HDT), and dithiothreitol (DTT). Signal in absence of the target, blank signal (white bars), and in the presence of the target at 10 nM (grey bars). Reprinted with permission from Ref. [57]. Copyright 2016 John Wiley and Sons.

Lopez-Ruiz and co-workers studied an impedance biosensor incorporating a ternary SAM composed of a thiolated aptamer, 1,6-hexanedithiol, and MCH [57]. These authors observed that the sensor stability depends on the Au surface roughness, functional groups on the SAM components, buffer employed during storage, and gaseous storage environment. They also conclude that the effectiveness of 1,6-hexanedithiol in a ternary SAM reflects its lack of additional reactive functional groups. Figure 3 illustrates their detailed results for the addition of short-chain diluents mercaptopropionic acid (A,B), 1,6-hexanedithiol (C,D), and dithiothreitol (E,F) within a ternary SAM that also includes thiolated aptamer and MCH for gluten (A,C,E) and peanut (B,D,F) genosensors [57]. 

Miodek, Estrela and co-workers reported that optimal sensor performance and stability during addition of different ternary components to the thiolated capture probe and MCH depend on the concentration, nature of the functional groups present, and chain length of the third component [55]. In addition, they noted that the resistance to non-specific adsorption of sensor electrodes created using ternary SAMs degrades after 28 days of storage.

Yang, Yu, and co-workers recently reported a multiplexed amperometric biosensor platform using a different immobilization chemistry, the cycloaddition reaction of three alkyne-modified DNA and aptamer probes with azido-terminated Au-thiol SAMs [59]. They report that with storage in a centrifuge tube at −20 °C, no detectable sensor loss occurred after one week, 95% of the initial response was retained after one month, and 90% remained after three months. The authors do not comment on the reasons for their enhanced storage stability. They report standard deviations in the amperometric response of their three electrodes of 1.1%, 1.5%, and 3.4%. Also noteworthy is their use of two sensor regeneration protocols within the same device, deionized water rinsing for DNA rehybridization and 4.0 M guanidine HCl incubation to disrupt aptamer–protein binding [59].

Results like those discussed above for ternary SAMs may be impossible for protein-based probes, which are larger and more complex and form less well-ordered SAMs. Biomolecule immobilization onto Au-thiol SAMs through the ω-functionalization is relatively easy to perform and employs readily available chemical reagents. For applications that involve creation and immediate sensor usage in a microfluidic device, Au-thiol chemistry provides the simplest and most direct route. Since it is widely studied and reasonably well understood, Au-thiol chemistry also provides a useful platform to study the physical chemistry of surface-immobilized biomolecules. 

Unfortunately, Au-thiol chemistry lacks adequate stability for most biosensor applications [48,60]. Biosensor stability can range from days to weeks, depending on the nature of the functional groups involved in the Au-thiol SAM, the specific biomolecules that are immobilized, and the storage conditions, particularly the presence of oxidizing agents and ultraviolet light. The fundamental limitation on Au-thiol chemistry for biomolecule immobilization is the weakness of the Au–S bond. Experimental estimates for the binding energy of thiol compounds in Au-thiol SAMs are ~30 kcal/mole [24,61], although gas phase studies imply binding energies of up to 50 kcal/mole [24]. Some researchers suggest that the interaction energy increases by ~2 kcal/mole for each additional CH_2_ group [24], but others report that the interaction energy does not depend on chain length [61]. Considerably lower values of 10–20 kcal/mole are reported for Au–S bond strength of individual molecules without formation of a two-dimensional, semi-crystalline SAM [62,63]. 

### 2.2. Multidentate Thiols

**Figure 4:**
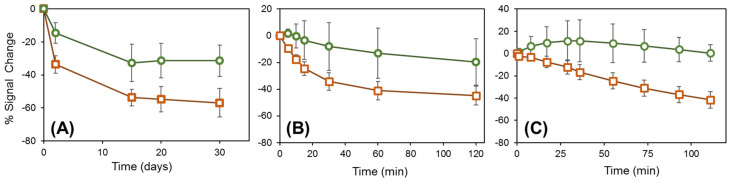
Percentage signal change for the tridentate thiol TA-1 (circles) and the monodentate thiol MA-1 (squares) probe films when (**A**) stored in PBS buffer at 4 °C for 30 days; (**B**) immersed in PBS buffer at 40 °C for 2 h; and (**C**) immersed in PBS buffer containing 2 mM dithiothreitol (DTT) at room temperature. Reprinted from Ref. [67].

A direct method to increase the stability of Au-thiol self-assembled monolayers (SAM) is to increase the number of Au–S bonds through the use of multidentate thiols, with multiple thiol groups on each linker molecule [64]. Miyahara and co-workers immobilized DNA atop Au by amide bond formation to a tridentate thiol containing three sulhydrylbenzyl moieties ω-functionalized with carboxylate groups [65]. This substantially improved the interfacial stability at elevated temperature (50–90 °C), as determined by fluorescence labeling and intensity measurements, but storage stability was not investigated [65]. A bidentate thiol, 16-[3,5-bis(mercaptomethyl)phenoxy]-hexadecanoic acid (BMPHA), was recently reported by Lee, Suni, and co-workers for antibody immobilization through amide bond formation to the ω-carboxylate groups of BMPHA, and the antibody film was regenerated daily using a mild chaotropic agent, 0.2 M KSCN, and interrogated by electrochemical impedance spectroscopy [66]. The bidentate thiol increased the sensor lifetime relative to a monodentate thiol from 10 to 20 days. Most recently, a tridentate thiol compound was used by Mount, Bradley and co-workers to immobilize and study a redox-labeled peptide within a mixed SAM that also contains 2,2′-(ethylenedioxy)diethanethiol (PDT) [67]. The peak current obtained from square wave voltammetry was compared between immobilization through a monodentate and tridentate thiol. While the peak current using the monodentate thiol linker decreased by almost 60% after 30 days of storage in pH 7.4 PBS buffer at 4 °C, the peak current using the tridentate thiol linker decreased only 30%. This is illustrated in Figure 4A, where the green circles show the% signal change with storage time for the tridentate thiol, and the orange squares show the same results for the monodentate thiol [67]. 

The use of dithiocarbamate (DTC) as a bi-functional reagent for Au biofunctionalizaton can be regarded a specific example of bidentate chemistry. DTC complexes are formed by solution phase reaction of amine groups atop biomolecules with CS_2_, and these DTC complexes then spontaneously self-assemble atop an Au surface. This approach was first reported by Liu and co-workers using DNA [68]. DTC complexes atop Au are widely described as involving formation of an S–Au–S bond. However, the energetic stabilization relative to a single Au–S bond is estimated to be only 13%, and the packing density atop Au is also reduced [69]. Several research groups have studied the stability of DNA [70,71,72], antibody [73], and laccase enzyme [74] immobilized atop Au substrates through DTC. The most detailed studies from Liu, Zho and co-workers report ssDNA immobilization onto an Au electrode through DTC, followed by detection of complementary DNA using differential pulse voltammetry (DPV) [70]. DNA-coated Au electrodes were regenerated by heating in hot water (80 °C) for 10 min., with only 8% of the signal lost after 11 regeneration cycles. In addition, DTC–DNA refrigeration storage showed minimal loss of DTC–DNA after one month [70]. 

While energetic stability is gained through the use of multiple Au–S bonds, some energetic stability is sacrificed through the loss of van der Waals interactions between adjacent hydrocarbon chains in a semi-crystalline, two-dimensional SAM. In addition, disulfide bond formation complicates synthesis and separation of multidentate thiols [75]. Many such reagents are not commercially available or are only available at high cost. 

### 2.3. Other Metals

Formation of SAMs atop other noble metals, including Ag, Cu, Pd, Pt, Hg and their alloys, has also been reported [24]. However, attachment of biomolecules to SAMs atop other noble metals has not been studied in detail. Recent evidence was reported that Pt-S bonds might be stronger than Au–S bonds [76], but this has not been thoroughly explored. 

## 3. Other Noble Substrate Materials

Some requirements appear to be contradictory when considering substrate materials for covalent biofunctionalization. For example, Au is biocompatible due to its low reactivity, so it does not leach out into test matrices or react detrimentally with tissue or body fluids. On the other hand, the low reactivity of Au makes stable chemical attachment of biomolecules quite challenging. This section considers other relatively noble substrate materials such as C, Si, and Pt where biomolecule attachment is also challenging. 

### 3.1. Carbon

Carbon is the most obviously biocompatible substrate material, since all life on Earth is carbon-based. Carbon is also widely used in electrochemical technology, including as cathodes and anodes in batteries and supercapacitors, anodes in electrodeposition cells, and for anodic stripping voltammetry. For biosensor electrodes, electrically conductive forms of carbon include boron-doped diamond, glassy carbon, amorphous carbon films, graphite, and carbon paste. Glassy carbon is a pyrolyzed material that contains mainly sp^2^-hybridized carbon chains and can be thought of as a mixture of graphite and a glassy ceramic. Carbon paste electrodes contain graphite powder mixed with a liquid paste.

According to recent reviews, the most widely utilized method for biomolecule immobilization atop carbon electrodes is thermal or ultraviolet activation of alkene or alkyne insertion reactions, which are otherwise quite slow [30,31]. These bifunctional reagents have alkene or alkyne groups at one end and chemically reactive groups such as amides, carboxylic acids, ethers, esters, or halogens at the other [30,31]. As for Au-thiol self-assembly, the most common approach is to use ω-carboxylate groups for amide bond formation using carbodiimide chemistry.

**Scheme 1:**
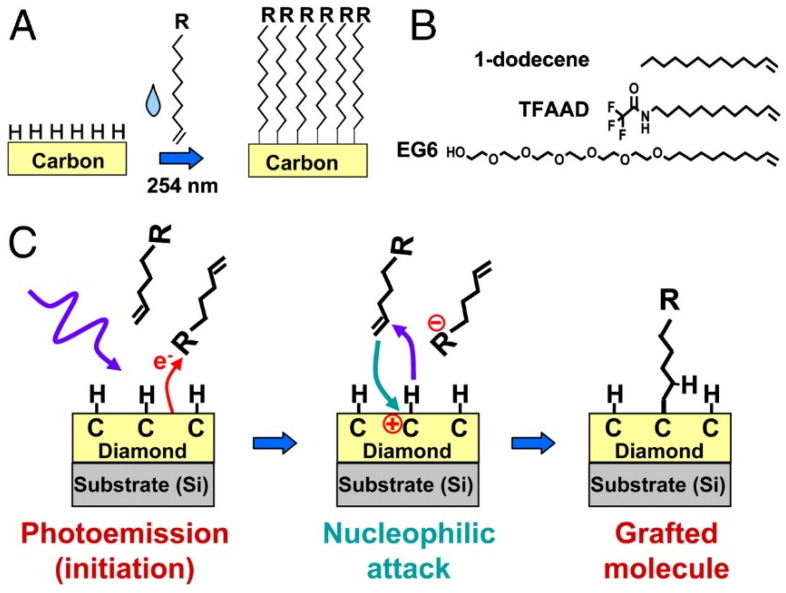
(**A**) Photochemical grafting of alkenes to H-terminated surfaces of carbon. (**B**) Molecules employed. (**C**) Simplified mechanism of photochemical grafting to surfaces of diamond and other forms of carbon. Reprinted with permission from Ref. [80]. Copyright 2011 National Academy of Sciences.

**Figure 5:**
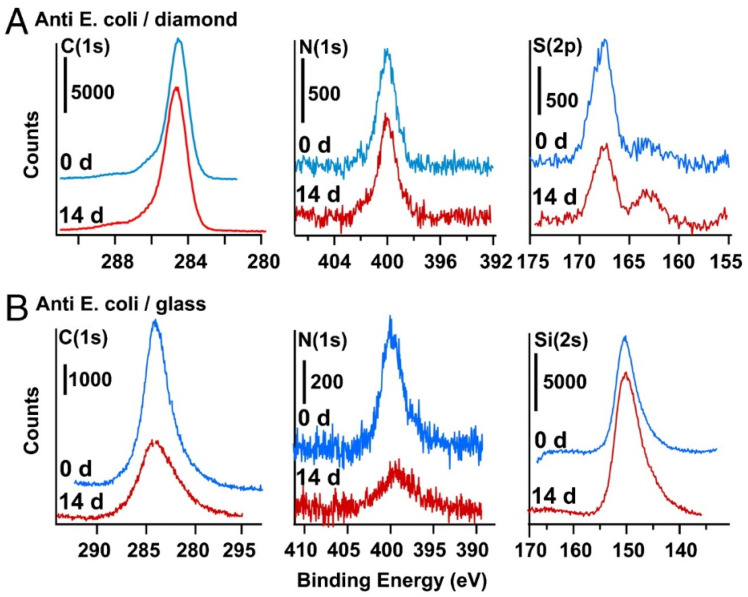
XPS data for surface composition of C, N, S and Si, illustrating stability of *E. coli* antibody covalently grafted to amine-terminated diamond (**A**) and amine-terminated glass substrates (**B**), before (blue) and after (red) storage for 14 days in PBS buffer at 37 °C. Reprinted with permission from Ref.[80]. Copyright 2011 National Academy of Sciences.

The most detailed studies that demonstrate high stability of immobilized biomolecules were published by Hamers and co-workers [77,78,79,80]. In one report, a nanocrystalline diamond thin film was exposed to a mixture of 10-aminodec-1-ene and 1-dodecene, with the latter reagent employed as a spacer, followed by photochemical activation of the alkene insertion reaction, as shown in Scheme 1 [80]. The exposed amino groups were then reacted with glutaraldehyde via reductive amination to yield an aldehyde-terminated surface, followed by incubation in antibodies to *Escherichia coli* O157:H7. These antibody films were tested for six cycles of cell capture and antibody regeneration using 0.1 M glycine-HCl buffer (pH 2.1), with no discernible loss of capture efficiency [80]. In addition, the antibody half-life for storage in pH 7.4 PBS buffer at 4 °C could not be observed during a 28-day test. Figure 5 illustrates the improved lifetime of the antibody coating atop thin film diamond relative to that atop glass [80]. The X-ray photoelectron spectroscopy (XPS) peaks for C and N in Figure 5 decline substantially over a 14-day period atop glass (pane B), but remain relatively constant atop thin film diamond (pane A).

In these studies [80], Hamers and co-workers provided evidence for an increase in both biological activity and stability upon immobilization via glutaraldehyde crosslinking. While glutaraldehyde crosslinking can be employed for biofunctionalization, its general applicability may be limited by its complexity and the lack of detailed mechanistic understanding. Aqueous glutaraldehyde solutions exhibit complex equilibria between multiple reactive monomeric and polymeric species, with the reactivity of the different species varying with pH [81,82]. In addition, these different species can react with multiple protein functional groups, including amines, thiols, phenols, and imidazoles [81,82]. 

### 3.2. Silicon

At first glance, silicon appears quite promising as a substrate material for biofunctionalization of electrochemical sensors. Silicon is directly beneath carbon in Group 14 of the periodic table, so it has similar properties, including biocompatibility and reasonable electrical conductivity when degenerately doped. In addition, silicon-based biosensors can be easily incorporated into microelectronic circuitry. The most serious problem for electrochemical biosensors with Si substrates is spontaneous oxidation to SiO_2_, which is an electrical insulator. Si-based electrochemical biosensors require removal of the SiO_2_ native oxide with HF to form H-terminated Si, followed by biofunctionalization and surface passivation to prevent oxide reformation. H-terminated Si(111) is more stable than other surface orientations, and Si(111) yields more ideal self-assembled monolayers (SAMs) when the native oxide is removed with buffered HF etchant [32]. 

The most widely utilized method for functionalization of silicon (like carbon) electrodes is thermal or photochemical activation of alkene or alkyne insertion reactions, as recently reviewed [32,33]. Some researchers have concluded that formation of dense polymer films atop Si is limited by the large areal density of alkyl groups relative to the atomic spacing between Si atoms on the surface [32]. All-trans alkyl groups occupy ~0.20 nm^2^ when perfectly aligned to the surface, but an even larger area when tilted. The optimal packing density is reduced even further for a functional reagent with ω-carboxylate or other groups for protein immobilization. The research group from Physique de la Matière Condensée, École Polytechnique at CNRS reports that the maximum surface coverage of a SAM atop Si is 50–60%, and that this is inadequate to prevent gradual Si oxidation, which eventually displaces the polymer film [32]. Gooding and co-workers suggested an approach to circumvent this problem using 1,8-nonadiyne, which has alkyne groups at both ends, to form fully acetylenated SAMs with a smaller areal footprint atop Si(100) [83]. For 1,8-nonadiyne SAMs atop Si(100), no oxide formation to within the resolution of XPS could be detected atop Si(100) after 1000 potential cycles between −100 and +800 mV (vs. Ag/AgCl), or after storage for two months in an Ar atmosphere at 4 °C [83]. They reported an XPS resolution or approximately 0.07 SiO_x_ monolayers. However, subsequent attachment of biomolecules to acetylenate groups atop Si(100) does not appear to have been reported. 

Despite the complications of oxide regrowth atop biofunctionalized Si electrodes, several research groups have reported improved durability relative to Au-thiol SAMs [84,85,86]. Radhakrishnan and Suni demonstrated that antibodies to the allergenic peanut protein Ara h 1 could be regenerated daily using 200 mM KSCN + 10 mM HF as a chaotropic reagent, with a sensor lifetime of 30 days atop n-type degenerate Si(111) but only 15 days atop Au-coated glass slides, with amide bond formation to carboxylate-terminated SAMs on both surfaces [84]. Compared to n-type or p-type Si, degenerate Si has the advantages of a higher electrical conductivity and a simpler equivalent circuit for detection by electrochemical impedance spectroscopy [84]. Sam and co-workers reported immobilization of tyrosinase onto porous Si via amide bond formation to an undecylenic acid SAM [85]. They reported that a pyrocatechol ultraviolet (UV)-visible absorption sensor retained 80% of its initial activity after six months of storage at 4 °C. They also obtained good reproducibility, with a standard deviation of 7.2% from four optical sensor measurements [85]. However, porous Si is mechanically unstable due to its brittleness and susceptibility to oxidation. This well-known shortcoming of porous Si may limit its applicability to biosensors [87]. Allongue, Gouget-Laemmel and co-workers demonstrated that a surface-immobilized aptamer to ochratoxin A atop n-type Si(111) can be used to capture the antibody to ochratoxin A [86]. This antibody was recycled five times with a regeneration buffer containing 10 mM tris + 120 mM NaCl + 20 mM CaCl_2_ + 5 mM KCl at pH 8.5, as monitored by Fourier transform infrared (FTIR) spectroscopy in attenuated total reflection (ATR) geometry. The aptamer to ochratoxin A was immobilized atop Si by photochemical hydrosilylation to form a surface monolayer, followed by amide bond formation using carbodiimide chemistry [86].

A novel method was recently reported by Darwish and co-workers for direct formation of Si–S bonds by the exposure of H-terminated Si to thiolated compounds, or even more rapidly by disulfide bond reduction [88,89]. The mechanism is not yet understood, but appears to require the presence of moderate amounts of oxygen, since this process works poorly when oxygen is either completely absent or present at high concentrations. However, a dense SiO_2_ layer does not appear to form, since the surface remains electrochemically active [88,89]. Protein immobilization was demonstrated by these authors using the model protein azurin that contains a disulfide bond at one terminus and a buried copper metal center at the other end of the protein [89]. The stability of biomolecules immobilized atop Si using this approach has not yet been ascertained. 

### 3.3. Platinum

Platinum is one of the most noble metals and is expected to be biocompatible. However, few studies have been published on the stability of Pt biofunctionalization. As mentioned in Section 2.3, Pt–S bonds were recently hypothesized to be stronger than Au–S bonds [76]. Several groups recently studied biomolecule immobilization atop Pt, including antibody binding through amide bond formation to a Pt-thiol SAM [90], direct formation of a Pt–S bond to a thiolated aptamer [91], and formation of a ternary SAM that includes a thiolated peptide, diethanethiol, and 6-mercapto-1-hexanol [92]. McLamore, Gomes and co-workers studied an impedance biosensor format with aptamer regeneration using 2.0 M NaOH [91]. They achieved five cycles of *Listeria* spp. detection, after which the sensor became unstable, but they did not study long-term storage stability. Bradley, Mount and co-workers reported that after storage for one week in pH 7.4 PBS buffer at 4 °C, the square wave voltammetry (SWV) signal for trypsin detection declined less for Pt microelectrodes (20%) than for Pt macroelectrodes (40%) [92]. They also reported improved sensor reproducibility for three Pt microelectrodes relative to three Pt macroelectrodes [92].

## 4. Transition Metal Dichalcogenides 

Two-dimensional (2D) transition metal dichalcogenides (TMDs) such as MoS_2_, WS_2_, MoSe_2_ and WSe_2_ that are 1–3 monolayers thick exhibit direct band gaps, attracting significant recent attention for possible use in electronic [93,94] and optoelectronic devices [95,96]. Bulk TMDs have an indirect bandgap and a distinctive layered hexagonal structure with strong covalent bonds within each layer, but only weak van der Waals bond between layers, attracting attention for possible use in battery and supercapacitor electrodes [97,98]. Recent excitement regarding TMDs extends to their possible use as biosensor substrate materials, where their advantages include high natural abundance, chemical stability, easy preparation methods that can be scaled up for mass production, and good biocompatibility [99,100]. In addition, 2D TMDs exhibit rapid heterogeneous electron transfer, high density of electronic states, and high fluorescence intensity [99,100].

Due to their chemical inertness, poor adhesion, and low electrical conductivity, many biosensor studies employ substrate materials that are composites of TMDs with other materials such as Au nanoparticles or conductive polymers [101]. Such studies will not be discussed here, since the biomolecular immobilization atop such composites is difficult to understand. For example, attributing the observed stability or instability to specific materials is difficult. Synergistic effects may exist, but will be difficult to quantify. Thin-film TMDs as pure compounds are not commercially available, except as special orders. 

Most biosensor studies to date have utilized physisorption for biomolecule immobilization atop TMDs [102], but this is likely not robust enough for commercial applications. Most methods for chemical and biomolecular functionalization of TMDs exploit the chemical reactivity of sulfur vacancies and the corresponding exposed molybdenum atoms [103,104]. Thiol compounds are particularly reactive with sulfur vacancies and have been studied extensively for defect repair, dopant introduction, and functionalization of TMDs [103,104]. A trade-off typically exists between surface repair, where S-C bonds are broken within thiol compounds, and molecular chemisorption, where chemical bonds within thiols are preserved [103]. Similar energetic tradeoffs exist during commercial hydrodesulfurization (HDS), which utilizes TMD catalysts for cyclical adsorption and desorption of sulfur compounds for their removal from hydrocarbon streams [105]. 

Exploiting the affinity of TMDs for thiol compounds, Sabherwal, Deep and co-workers reduced the disulfide bounds in the hinge region of prostate specific antigen (PSA) antibodies using 2-mercaptoethylamine-HCl, and bound the thiolated antibody fragments to MoS_2_ nanosheets in a field effect transistor (FET) biosensor format [106]. Suni and co-workers reported binding of thiolated antibody fragments to electrodeposited MoS_2_ and Cu-doped MoS_2_ thin films using a different reducing agent, tris (2-carboxyethyl) phosphine (TCEP) [107]. Thiol reactivity of TMDs was also used by Prasad and co-workers for a bifunctional reagent, dithiobis [succinimidyl propionate] (DSP), containing an internal disulfide bond, which was chemisorbed atop MoS2 nanosheets [108]. Cortisol antibody was then bound to the DSP crosslinker, and tested in an impedance biosensor format. 

MoS_2_ functionalization with carboxylate groups, and subsequent attachment of biomolecules through amide bonds using carbodiimide chemistry, has also been reported [109,110]. Exposure of MoS_2_ to chloroacetic acid, where chlorine atoms bind to sulfur vacancies created using ultrasound, was recently reported by Chiu and Lin to create carboxylate-terminated MoS_2_ [109]. Amide bond formation to bovine serum albumin (BSA) was then used to create a surface plasmon resonance (SPR) biosensor [109]. Amide bond formation was also utilized by Gondran, Holzinger and co-workers to immobilize tyrosinase atop carboxylate-functionalized WS_2_ nanotubes [110]. For those studies, the authors utilized a mixture of O-alkylating 2-bromoacetic acid and silver acetate in anhydrous dimethylformamide (DMF) to fabricate carboxylate-functionalized WS_2_ nanotubes through a Vilsmeier–Haack (VH) type mechanism [111]. 

None of the publications cited in this section report detailed studies of sensor stability, storage lifetime, or regeneration. While the use of MoS_2_ and other TMDs as substrate materials for immobilization of biomolecules is quite intriguing, future research needs to address these issues. Other issues that must be addressed include the electrical conductivity of TMDs and the ability to make the ohmic contacts needed for electrochemical biosensors. Pure MoS_2_ is an n-type semiconductor whose bulk resistivity is high but is not clearly specified in the academic literature. Substitutional doping for Mo cations is widely believed necessary for commercial usage but is still an active research problem [112,113,114,115]. In addition, obtaining ohmic contacts to TMDs such as MoS_2_ is challenging [116]. 

## 5. Electrically Conductive Metal Oxides

As noted above, many relatively noble elements (i.e., C, Pt, Si) are both biocompatible and electrically conductive, but due to their chemical inertness they are difficult to biofunctionalize. In theory, electrically conductive metal oxides should be easier to chemically modify, but unfortunately most metal oxides are poorly conductive. 

Silanization is often employed for biomolecule immobilization atop oxides such as SiO_2_ and various forms of glass. Silanization involves cleaning and surface activation to maximize the surface concentration of hydroxyl (OH) groups, formation of an alkoxysilane polymer atop the surface through Si–O–Si bond formation, rinsing to remove unbound silane, and biomolecule attachment to the silanized layer through an appropriate functional group [34,35]. The most commonly used alkoxysilanes for biofunctionalization are (3-aminopropyl)-triethoxysilane (APTES) and 3-glycidyloxypropyltrimethoxysilane (GOPTS). 

While silanization has the potential to create biointerfaces that are more stable than those obtained using Au-thiol chemistry, silanization has some shortcomings that suggest it may not be generally applicable to biofunctionalization. Silanization protocols are not well standardized, so results often vary with time, temperature, and solvent, and the choice of silane reagents and concentrations varies [34,117]. In addition, silanization depends critically on the hydration state, so the precursor may copolymerize in the presence of trace water, forming aggregates and disordered surface layers on the substrate. The silane layer may also be unstable in aqueous media, undergoing hydrolysis of siloxane bonds [34,117]. 

The most common alternative to silanization chemistry for biofunctionalization of metal oxides is the use of phosphonic acid or phosphonate coupling chemistry [36]. Phosphonate bifunctional reagents form iono-covalent bonds between PO_3_ groups and metal atoms at one end, with ω-functional groups at the other end for biofunctionalization. Thus the use of phosponate chemistry for biofunctionalization of metal oxides is similar to methods that utilize Au-thiol SAMs in that the linker chemistry should be limited to one monolayer thickness. Phosphonate chemistry is also applicable to a broader range of metal oxides than silane chemistry. 

### 5.1. Infdium Tin Oxide (ITO)

Tin-doped indium oxide, commonly known as indium tin oxide (ITO), is a widely commercialized transparent conducting oxide that has both high electrical conductivity and optical transparency, and can be easily deposited as a thin film. The use of ITO within biosensors has been recently reviewed [37]. The most common methods for biofunctionalization of ITO are through silanization and phosphonate chemistries. Phosphonate chemistry atop ITO can be complicated by the dependence of SAM quality on the source of ITO thin films, which may be polycrystalline or amorphous, have varying surface roughness and grain diameter, and even varying composition across the surface [118]. SAM formation also depends upon the solvent chosen [119].

Sezgintürk and co-workers recently reported detailed studies of stability, storage, and regeneration for biofunctionalization of ITO electrodes using silanization chemistry [120,121,122]. They immobilized antibodies using carbodiimide chemistry to form amide bonds between amino groups on the antibody surface and carboxylate groups atop their silanized films, which are created using carboxyethylsilanetriol (CTES) or 3-cyanopropyl trimethoxysilane (3-CPTMS) [120,121]. In one study they report an impedance biosensor for the transcription factor SOX2, which is involved in cancer signaling, including detailed studies of sensor stability, storage lifetime, and antibody regeneration using 0.1% HCl as a chaotropic reagent [120]. They found that after eight regeneration cycles, the sensor retained 22% of its sensitivity, as monitored by the charge transfer resistance obtained from impedance measurements. They also studied the storage stability of their impedance biosensors by evaluating this weekly over a 10-week period during dry storage at 4 °C. After nine weeks, their sensors retained 79% of their initial activity. In addition, they compared the calibration curves of 10 different sensors prepared under the same conditions. The relative standard deviation of the slope (intercept) of the linear calibration curves was 4.3% (5.1%) [120]. 

Lobo-Castanon and co-workers recently reported detection of *Salmonella enterica* by differential pulse voltammetry atop silanized ITO electrodes [123]. The ITO surface was first silanized by APTES, and then thiolated DNA specific to *Salmonella* were covalently attached to amino groups in APTES using sulfosuccinimidyl-4-(*N*-maleimidomethyl) cyclohexane-1-carbonate. They reported 9-month storage stability for dry storage of DNA-coated ITO electrodes at 4 °C, following rehydration with phosphate buffer solution (PBS). 

**Figure 6:**
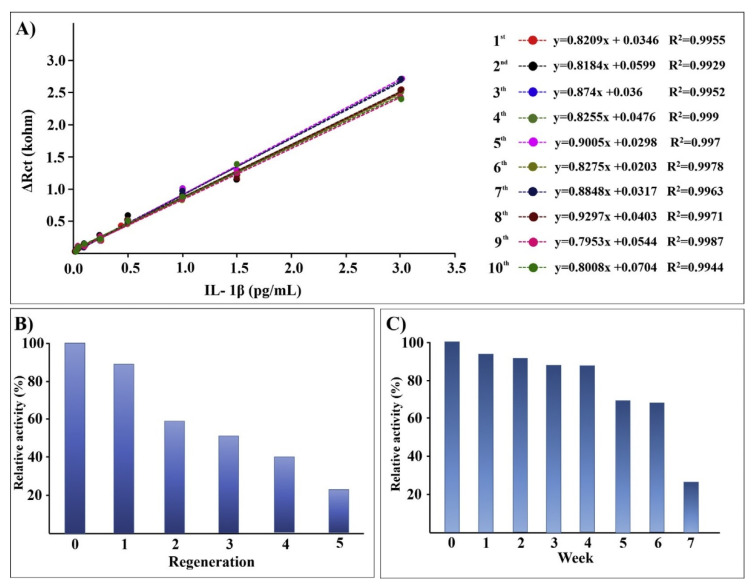
Results for reproducibility (**A**), regeneration (**B**), and storage stability (**C**) for impedance detection of interleukin 1β (IL-1β) atop antibody-coated indium tin oxide (ITO). Reprinted with permission from Ref.[125]. Copyright 2018 Elsevier Inc.

Aydun and Sezgintürk also reported detailed studies of antibody immobilization onto ITO through amide bond formation to carboxylate-terminated SAMs created from 6-phosphonohexanoic acid [124,125]. These ITO electrodes were employed for impedance detection of interleukin-1β (IL-1β), a key inflammatory cytokine secreted upon infection, cellular injury, or as an immune response. They reported a standard deviation of 5.4% for 80 electrodes for the charge transfer resistance from impedance detection. The sensor electrodes retained 26% of their signal after dry storage for 10 weeks at 4 °C, and antibody regeneration with 0.1% HCl was successful over five cycles, as illustrated in Figure 6 [125]. 

### 5.2. Titanium Dioxide (TiO_2_)

Titanium dioxide (TiO_2_) seems promising as a substrate materials for biofunctionalization for several reasons. Titanium metal is widely utilized in biomedical implants, in part because thin barrier layer of TiO_2_ is expected to form in vivo, blocking Ti oxidation as well as Ti^4+^ leaching outwards. TiO_2_ is a widely abundant materials used in paints, sunscreen, and pigments. Silane and phosphonate chemistries can both be applied to TiO_2_ biofunctionalization.

Covalent attachment of collagen and fibronectin to TiO_2_ has been widely investigated using both silane and phosphonate chemistries to alter the chemical and morphological properties of the surface of biomedical implants, and to improve osseointegraton [126,127,128,129]. While short-term interfacial stability has been reported in some cases, no studies of stability have been reported in a biosensor format. 

Indeed, few electrochemical biosensors have been reported that incorporate Ti or TiO_2_ substrates. Mantzila and Prodromidis studied the use of anodized Ti as a biosensor substrate with signal transduction by electrochemical impedance spectroscopy [130,131]. TiO_2_ films 30–86 nm thick were grown in 1.0 M H_2_SO_4_, hydroxylated in 0.5 M NaOH, silanized in APTES, crosslinked with glutaraldehyde, and incubated in avidin. While biotinylated dextran could be detected, the reported impedance values were in the MΩ range due to the low conductivity of the TiO_2_-polymer-protein film stack. Other recent reports of electrochemical biosensors atop TiO_2_ employ composite electrodes to increase the electrical conductivity. 

The main obstacle to the use of Ti-TiO_2_ as an electrode for biofunctionalization is the high electrical resistivity (>10^8^ Ω-cm) of TiO_2_, which is a wide bandgap (3.0–3.2 eV) semiconductor. Nb dopants, which substitute for Ti in the crystal lattice, can substantially increase the electrical conductivity of TiO_2_ [132], but may cause the TiO_2_ crystal structure to become unstable when used for electrochemistry [133]. Recent reports of sputtered Nb-doped TiO_2_ films with a resistivity of 630 × 10^−6^ Ω-cm [134,135] and Nb dopant levels up to 33% [135] appear to be promising for future biofunctonalization of TiO_2_ electrodes. However, the electrical conductivity of Ti/TiO_2_ is even further reduced when coated with a biofunctional polymer film. The widespread usage of Ti within biomedical implants makes TiO_2_ a promising biosensor substrate material should electrically conductive TiO_2_ thin films become widely available. 

## 6. Electrically Conductive Polymers

Electrically conductive polymers such as polyaniline, polypyrrole, and polythiopnene have attracted significant research attention for possible applications to sensing and biosensing as well as to energy storage and conversion, including batteries, supercapacitors, solar cells, and fuel cells [136]. Recent reviews indicate that many biofunctionalized electrodes based on conducting polymers contain composites of these polymers with carbon nanotubes, gold nanoparticles, MoS_2_, and other materials [136,137]. As discussed above, this makes understanding the contribution of different materials to the stability of biofunctionalization difficult. Biosensors that utilize electrically conductive polymer substrates have been recently reviewed [38]. In addition, several recent publications studied sensor stability, storage, and/or sensor regeneration for conducting polymers alone (without conductive additives) that are biofunctionalized with proteins [138,139,140], DNA [141], and bacteriophage [142]. 

Xu and co-workers reported room temperature anodic deposition of poly(thiophene-3-acetic acid) (PTAA) thin films from an electrolyte containing 0.5 M thiophene-3-acetic acid in the ionic liquid 1-butyl-3-methylimidazolium tetrafluoroborate, commonly known as Bmim-BF_4_ [138]. PTTA films were grown for 90 s at 2.2 V vs. SCE, but the film thickness was not reported. Glucose oxidase was subsequently immobilized atop PTTA by amide bond formation between exposed carboxylate groups on the PTTA film and exposed amino groups on the protein surface. The authors reported a decline of only ~2% after 28 days of storage in pH 7.5 PBS buffer at 4 °C, as determined by amperometric detection of glucose [138].

**Table 1:** Properties of different electrode substrate materials that have been biofunctionalized.

Material	ρ (Ω-cm)	Functionalization Chemistry	Advantages	Disadvantages	Key References ^2^
Au	2.44 × 10^−6^	Au-S bond	Simplicity, Cost, ULSI ^3^ compatible	Poor storage stability	[54,55,56,57,58,59,65,66,67,70,71,72,73,74]
C	10^14^ (diamond) 10^−2^–10^2^ (BDD) ^1^ 10^−1^–10^−4^ (graphite)	Alkene insertion	Biocompatibility	Lack of standard substrates, Slow immobilization chemistry	[77,78,79,80]
Si	2.3 × 10^5^ (undoped) 5 × 10^−3^ (degenerate)	Alkene insertion	ULSI compatible	Gradual SiO_2_ formation	[86,87,88,89,90,91,92,93,94]
Pt	1.06 × 10^−5^	Pt–S bond	ULSI compatible	Not well studied	
ITO	10^−4^	Phospnonate	Standard substrates and immobilization chemistries	Variation in substrate properties	[120,121,122,123,124,125]
TiO_2_	>10^8^	Phosphonate	Current use in biomedical implants	Very low conductivity	
MoS_2_ WS_2_	- ^4^ -	Thiol adsorption	Rapid technological advances	Lack of functionalization chemistry	
Polyaniline Polypyrrole Polythiophene	2 × 10^6^ (HBr doped) 0.01–0.5 (doped) 0.01 (doped)	Various	Probe incorporation during film growth possible	Lack of standard electropolymerization methods	[138,139,140,141,142]

^1^ BDD = boron-doped diamond. ^2^ Detailed studies of sensor stability, storage lifetime, and reproducibility. ^3^ ULSI = ultralarge scale integration. ^4^ Blank values indicate uncertainty.

Sarac and co-workers electrospun nanofiber mats composed of polyurethane and poly(m-anthranilicacid) (P3ANA) from a mixed solvent of dimethylformamide (DMF) and tetrahydrofuran (THF) [141]. The average nanofiber diameter of 546 nm was determined using Image J from scanning electron microscope (SEM) images. DNA was subsequently immobilized using carbodiimide chemistry to form amide bonds between carboxylate groups on the P3ANA surface and the probe DNA, which was amino-modified at the 5′ end and is specific to *Salmonella* spp. DNA hybridization was studied by impedance detection after 1, 7 and 30 days of sensor storage at 4 °C and retained ~93% of its initial response after 30 days [141]. 

Karoonuthaisirie, Thavarungkul and co-workers reported tyramine electropolymerization atop Au electrodes from an electrolyte containing 50 mM tyramine in 2.0 mM pH 7 PBS buffer and ethanol using cyclic voltammetry sweeps [142]. No film thickness was given for the polytyramine. M13 bacteriophage specific to *Salmonella* spp. was then crosslinked atop the polytyramine using glutaraldehyde. Their capacitive sensor was regenerated using 5.0 mM NaOH, and reuse up to 40 times was demonstrated with only 1% deterioration in capacitive response. In addition, measurements on six electrodes revealed a relative standard deviation in the capacitive response of only 1.1% [142]. 

**Figure 7:**
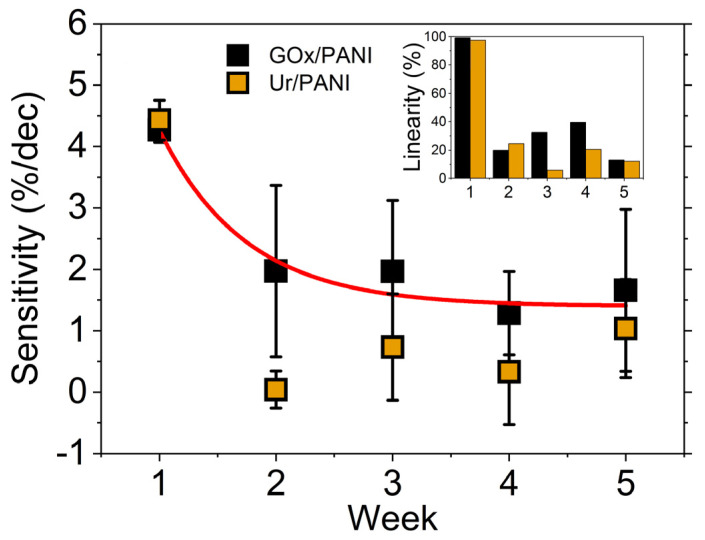
Sensitivity and linearity of glucose oxidase (GOx)/polyaniline (PANI) and uricase (Ur)/polyaniline (PANI) biosensors monitored for a five-week period. Reprinted with permission from Ref. [139]. Copyright 2020 Royal Society of Chemistry.

Detailed studies of biofunctionalization of conducting polymer electrodes were recently published by Mello and co-workers [139,140]. They incorporated glucose oxidase or uricase within polyaniline thin films by simply adding different concentrations of the soluble enzyme to their electropolymerization electrolyte, which also contains 0.1 M aniline and 0.1 M KCl. Unlike most studies discussed here, glucose oxidase or uricase is essentially part of a two-component composite film with polyaniline, which greatly simplifies biofunctionalization. These thin films are used as biosensors to detect glucose and urea through enzymatic production of different cations and anions (i.e., H^+^, OH^−^, NH_4_^+^). Detection of ions was compared using two different transduction methods, potentiometry and optical reflectance, and the sensor stability over a five-week period varied between the different formats [139,140]. However, potentiomeric detection of urea through OH^−^ production yielded stable results over a five-week period with sensor storage in Petri dishes at 4 °C. Figure 7 illustrates the variation in sensitivity and linearity over a five-week monitoring period for their optical reflectance biosensor.

## 7. Aryldiazonium-Based Electrochemical Reduction 

**Scheme 2:**
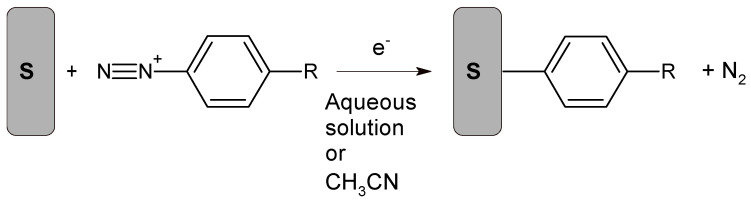
Electrografting of diazonium salts. Reprinted from Ref. [143].

Electrode biofunctionalization using aryldiazonium chemistry is currently an area of intense interest [143,144]. Aryldiazonium cations are reduced from an acetonitrile or acidic aqueous electrolyte to form a covalent bond to the substrate, with simultaneous N_2_ evolution, as illustrated in Scheme 2 [143]. Although the reactant is an aryldiazonium cation, the detailed reaction mechanism involves formation and subsequent facile reactions involving aryldiazonium radicals. Several reduction methods have been studied, including application of a cathodic electrochemical potential, addition of a chemical reducing agent, photochemical activation, and the use of reducing surfaces such as oxide-free metals or carbon that are simultaneously oxidized [143,144]. In some cases, the starting material is an aryldiazonium cation, often with a BF_4_^−^ anion, but in situ cation generation is considered attractive because some salts are unstable. Biofunctionalization typically occurs through reactive groups on the benzene ring within the aryldiazonium cation. 

The ease with which aryldiazonium cations can be reduced to form covalent bonds with a variety of substrate materials is a major advantage of this approach. However, the high chemical activity of aryldiazonium radicals also makes this reaction quite difficult to control, often yielding films that are either uneven or thicker than desired. For electrochemical biosensors, uniform and complete surface coverage with thin aryldiazonium layers is advantageous in order to minimize film resistance, but some evidence exists that the electrical conductivity increases as the film becomes thicker [145]. Methods to limit this method to monolayer formation include chemical scavenging of excess aryldiazonium, unconventional ionic liquids as electrolytes, bulky functional groups that inhibit further reaction, and organic protection-deprotection groups, as reported both in the recent literature [146,147,148,149,150] and in recent reviews [151,152,153]. 

Aryldiazonium chemistry is used most often to modify carbon substrates, but is also used frequently with gold substrates, and is in principle applicable to any substrate material [143,144]. However, some substrates such as MoS_2_ and other TMDs are less reactive, posing challenges. Wang and co-workers recently reported spontaneous chemical functionalization of MoS_2_, WS_2_, MoSe_2_, and WSe_2_ from solutions containing 4-nitrobenzenediazonium tetrafluoroborate, which is the most commonly used arydiazonium salt [154,155,156]. Chemical reaction was proposed to start at sulfur vacancies present at low concentrations even atop pristine MoS_2_ nanosheets [154], which are otherwise chemically inactive, as discussed in Section 4. Covalent bond formation to MoS_2_, WS_2_, MoSe_2_, and WSe_2_ is demonstrated by FTIR observation of C–S and C–Se vibrations [156]. Wang and co-workers demonstrate attachment of polyhistidine-tagged green fluorescent protein to MoS_2_ by amide bond formation between the aryldiazonium film and nitrilotriacetic acid (NTA) [154]. Methods to control aryldiazonium film thickness atop MoS_2_ have been studied by several groups [157,158,159]. 

Recent studies of stability and storage lifetime atop aryldiazonium films have been reported for immobilization of both proteins [160,161,162] and DNA [163,164]. Nazemi, Shams and co-workers used six potential cycles for nitrophenyl film deposition atop glassy carbon from 1.0 mM 4-nitrobenzenediazonium tetrafluoroborate and 0.1 M tetrabutylammonium tetrafluoroborate in acetonitrile [160]. They subsequently deposited layers of Fe_3_O_4_ nanoparticles, chitosan, and glucose oxidase using layer-by-layer electrostatic deposition. Glucose was detected by amperometry, and exposure to ultrasound in an acidic electrolyte had no effect on the current detected, demonstrating that glucose oxidase is tightly bound. When stored in PBS buffer at pH 7.0, the modified electrode retained 94.2% (50%) of its original glucose sensitivity after 2 (20) days [160].

Yanez-Sedeno and co-workers recently studied multiplexed detection of obesity-related hormones by differential pulse voltammetry at graphene electrodes onto which the diazonium salt of 4-aminobenzoic acid was grafted by repeated potential cycling [161]. Carbodiimide chemistry was subsequently used to form amide bonds between carboxylate groups on the film surface and antibodies to ghrelin and peptide YY. The storage stability of dual bioelectrodes prepared on the same day and stored at 4 °C in an unspecified solution was also tested. Over a 10-day period the voltammetric response was unchanged at both electrodes to within the measurement accuracy [161]. 

**Figure 8:**
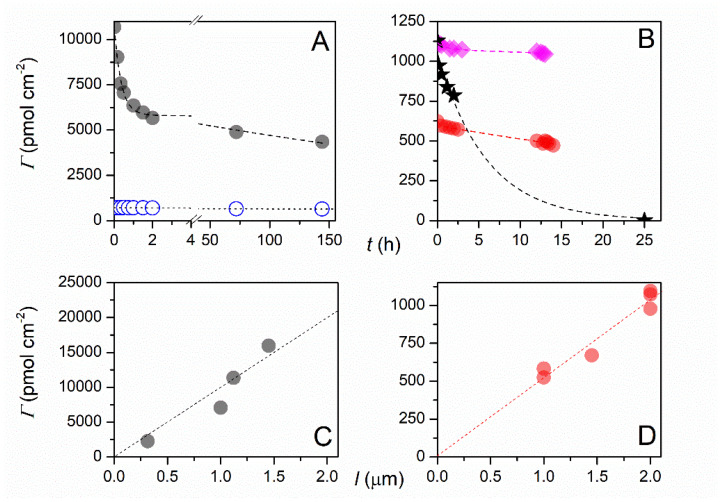
Surface coverage at 1 μm thick ITO electrodes as a function of soaking time in the electrolyte (0.3 M KCl + 0.1 M pH 7.5 HEPES buffer) for: (**A**) (●) ferrocene and (O) TAPP, (**B**) (●) rN6KHbI. In (**B**), the coverage data are compared to that obtained at a (♦) 2 μm thick rN6KHbI-ITO electrode and a (★) 1.45 μm thick ITO electrode. All experimental data are fitted to a bi-exponential decay function. (**C**,**D**) Surface coverage of (●) ferrocene and (●) rN6KHbI at ITO electrodes of varying film thicknesses l after 2–3 h soaking in the electrolyte. Reprinted with permission from Ref. [162]. Copyright 2019 Published by Elsevier B.V.

Balland and co-workers recently reported an optical H_2_S biosensor with recombinant hemoglobin I (rN6KHbI) from *Lucina pectinata* clams as the biomolecular probe [162]. These authors mixed 5 mM 4-aminobenzoic acid with 5 mM NaNO_2_ at 4 °C under Ar to generate 4-diazoniumbenzoic acid in situ, and then functionalized PVD-deposited ITO electrodes by potential cycling. rN6KHbI was immobilized by amide bond formation to the exposed carboxylate groups on the surface. The surface coverage of rN6KHbI was quantified by spectroelectrochemical measurements of the sharp Soret band at 407 nm [162]. Soaking the rN6KHbI-coated electrodes in 0.1 M HEPES buffer and 0.3 M KCl at pH 7 yielded a bi-exponential decline in the surface coverage of rN6KHbI, with rapid desorption of weakly bound protein but a much longer lifetime for tightly bound protein. They estimate the surface coverage (Г) of rN6KHbI as 477 pmol-cm^−2^ after 20 days storage in pH 7.5 HEPES buffer at 4 °C, down from its original value of 611 pmol-cm^2^ [162]. Pane A in Figure 8 illustrates the decline in surface coverage as a function of soaking time for ferrocene and porphyrin (TAPP) bound to ITO, whereas pane B illustrates the same results for recombinant hemoglobin (rN6KHbI) bound to two different ITO film thicknesses (red circles and purple diamonds). 

Li, Chuang and co-workers employed chronoamperometry to detect genetic fragments of New Delhi metallo-β-lactamase-coding gene at an Au electrode modified with an aryldiazonium film [163]. Acidic mixtures of two synthesized compounds, 3-((4-aminophenyl)-dimethylammonio)propane-1-sulfonate and *N*-(4-aminobenzoyl)-*N*′-(4-maleimidobenzoyl)-1,2-ethylenediamine, were used for in situ formation of diazonium cations, followed by potential cycling to grow a surface film. Thiolated DNA was then chemically reduced with tris (2-carboxyethyl)-phosphine hydrochloride to react with maleimide groups on the diazonium film. The stability of this sensor electrode was found to be similar to that of a comparable Au-thiol based sensor [163].

Mak and co-workers recently reported an impedance-based DNA sensor for the detection of anti-oxidant effects [164]. Amine-terminated DNA probes relevant to E908XWT breast cancer 1 were immobilized by amide bond formation using carbodiimide chemistry to a diazonium film deposited by potential cycling onto a screen-printed carbon electrode from an electrolyte containing 0.5 M H_2_SO_4_ and 5 mM 4-aminobenzoic acid tetrafluoroborate. The reproducibility of their DNA sensor was studied on five electrodes, and they reported relative standard deviation of 3.8%. After storage at 4 °C for three weeks, the impedance sensor charge transfer resistance was about 83% of its initial value [164]. 

The primary shortcoming of aryldiazonium salts for biofunctionalization is the high reactivity of the radicals formed during this process, which makes it difficult to control the film thickness and uniformity. This might be more problematic than widely recognized. Equation (1) in Section 1 illustrates the exponential increase in the charge transfer resistance (R_ct_) through an Au-thiol SAM with increasing thickness [53]. This reflects the exponential decrease in the rate of electron tunneling with increasing thickness. Therefore, the thinnest and most defective regions within a biomolecular film are the most heavily weighted during electrochemical measurements on macroelectrodes. Payne and Mauzerell reported that scanning electrochemical microscopy can be used to locate pinholes within aryldiazonium thin films [165]. Similarly, Jiang, Diao, Tong and co-workers used a combination of electrochemical methods to semi-quantitatively analyze defects within Au-thiol SAMs [166,167,168,169]. In summary, for some biofunctionalized electrodes, electrochemical measurements might only sample the most defective regions. 

For aryldiazonium films used within electrochemical biosensors, formation of relatively uniform monolayer films may be required to attain adequate reproducibility and sensor calibration. However, this problem may be ameliorated to some extent by the use of bovine serum albumin (BSA) and other blocking agents that may obscure film defects. 

## 8. Conclusions and Future Directions

The first electrochemical biosensors employed Au-thiol self-assembled monolayers (SAMs) created from linker molecules ω-functionalized with reactive groups (i.e., carboxylate) that can form covalent bonds to biomolecules. However, Au-thiol chemistry is fundamentally limited by the weak Au–S bond strength, suggesting that this approach is only useful for applications in which the sensor interface is used immediately, or for fundamental studies of biomolecules at interfaces. Although the use of multidentate thiols increases the number of Au–S bonds, in most cases the formation of a semi-crystalline 2D SAM is disrupted or weakened, losing some of the energetic gain from multiple Au–S bonds. Given the stability of Au and Pt, and their chemical inertness, future biofunctionalization research may not yield major new breakthroughs. 

Both carbon and silicon can be biofunctionalized with alkene or alkyne insertion reactions to form SAMs using bifunctional reagents that are ω-functionalized to form covalent bonds to biomolecules. Due to the widespread usage of Si within integrated circuits, and the availability of high-purity and atomically flat substrates, Si appears to be quite promising. However, Si biofunctionalization is currently limited by oxidation and gradual displacement of biopolymer films. On the other hand, carbon biofunctionalization is hampered by the limitations and availability of high-quality carbon substrates. Carbon electrodes include boron-doped diamond, glassy carbon, amorphous carbon films, graphite, and carbon paste. None of these is widely available with the following combination of desirable properties: high electrical conductivity, high elemental purity, controlled chemistry and morphology, and compatibility with high temperature or photo-activation of alkene-alkyne insertion. 

Metal oxides such as TiO_2_ or Sn-doped In_2_O_3_ (ITO) typically have greater chemical reactivity than noble substrate materials such as Au, Pt, Si and C. However, most widely available metal oxides are not sufficiently electrically conductive for use in electrochemical biosensors. Among metal oxides, ITO appears to be the best choice given its high conductivity, wide availability in thin film format, and its compatibility with phosphonate-based bifunctional self-assembly chemistry. However, ITO still depends to some extent on SAM formation, which is limited to nearly atomically smooth substrates. In addition, biofunctionalization of ITO may be complicated by variations in crystallinity, surface roughness, grain diameter, and surface composition of ITO thin films. 

Recent interest in biofunctionalization of electrochemical sensors has focused on transition metal dichalcogenides (i.e., MoS_2_), electrically conducting polymers, and in situ reduction of aryldiazonium salts, paralleling to some extent new research topics within the materials community. However, to date few approaches to biofunctionalization of MoS_2_ have been reported, and these focus mainly on the reactivity of sulfur vacancies. These approaches have yet to be studied in detail for their sensor stability and storage lifetime. The use of electrically conductive polymers is currently limited by the difficulty and reproducibility of polymer thin film deposition, and the long-term stability of film electrical conductivity. However, if biomolecules can be deposited simultaneously within the growing conductive polymer to form a composite film that already incorporates probe biomolecules, this is quite advantageous because bifunctional reagents are not needed for surface immobilization. The use of aryldiazonium film formation is quite attractive since this can be applied in principle to any conductive electrode. However, high reactivity of the radicals formed during this process make it difficult to control the film thickness and uniformity. The formation and control of monolayer films will likely continue to be a major focus of these efforts.
